# Simultaneous Improvement of Strength and Ductility of Dual-Phase Steel Processed by Multi-Step Cyclic Rolling and Intercritical Annealing

**DOI:** 10.3390/ma15186424

**Published:** 2022-09-16

**Authors:** Litao Liu, Bin Fu, Yanhui Guo, Liqun Wei

**Affiliations:** School of Materials Science and Engineering, Shanghai Institute of Technology, Shanghai 201418, China

**Keywords:** dual-phase steel, grain refinement, martensite distribution, fracture performance

## Abstract

In the present study, a multi-step (MS) cyclic rolling and intercritcal annealing process was proposed and applied for dual-phase (DP) steel. The MS process performed three times with 27% deformations and intercritical annealing, while the single-step (SS) process performed an 81% rolling, along with intercritical annealing. A microstructure with an average grain size of 3 μm and a martensite content of ~40% was obtained after MS treatment, which is similar to results obtained from the SS treatment. However, the distribution exhibits significant differences between the two different routes. A more homogenous distribution of ferrite–martensite was achieved after the multi-step compared with the single-step treatment. The yield strength of MS is slightly smaller than that of SS, while the ultimate tensile strength is better, which results in a decrease in yield ratio. Furthermore, the ductility was greatly improved after MS, which is mainly attributed to the uniform chain-like distribution of martensite.

## 1. Introduction

Dual-phase (DP) steel is the flagship of the advanced high-strength steel, which is thought to be the candidate steel for the weight reduction of components in the automotive industry due to its good comprehensive mechanical properties combined with good strength and ductility [1,2,3,4]. The mechanical properties of DP steel are greatly influenced by the differences between the ferritic and martensitic phases [5,6,7,8]. It has been found that the martensite volume fraction and the grain size of the ferrite affect the deformation mechanism, which in turn has an impact on the strengthening behavior of DP steels [9,10]. In addition, the morphology and spatial distribution of martensite were also found to have an effect on the tensile properties [11,12]. Normally, the strength increase due to the martensite volume fraction leads to a sharp decrease in ductility, which can be improved by a uniform martensite distribution.

Thermal cycling (repeated intercritical annealing with quenching) has been found to refine the band spacing between ferrite and martensite [13]. In the study of martensite distribution, it was also found that the delayed mechanism of crack growth at the ferrite–martensite interface promotes strain hardenability and enhances the ductility of the steel [14]. Farshchi prepared DP steels with chain-distributed martensite and verified the enhanced work hardening behavior based on factors such as work hardening rate and flexural strength ratio [15]. This suggests that multi-step annealing is beneficial for the properties of DP steel. However, to the knowledge of authors, the combination of rolling and thermal cycling routes is a relatively limited reported processing of DP steel, which is the main objective of the current work.

The main motivation of the present study is to investigate the effect of the combination of rolling and thermal cycling on the microstructure and mechanical properties of DP steel. Multi-step cyclic rolling and intercritical annealing were performed on DP steel. For comparison, the conventional method (single-step rolling and intercritical annealing), with the same total thickness reduction, was also used. The microstructure and mechanical properties were analyzed and discussed by considering the volume fraction, grain size, and distribution of ferrite–martensite in the DP steel processed by two different methods.

## 2. Materials and Methods

### 2.1. Materials Processing

**Figure 1:**
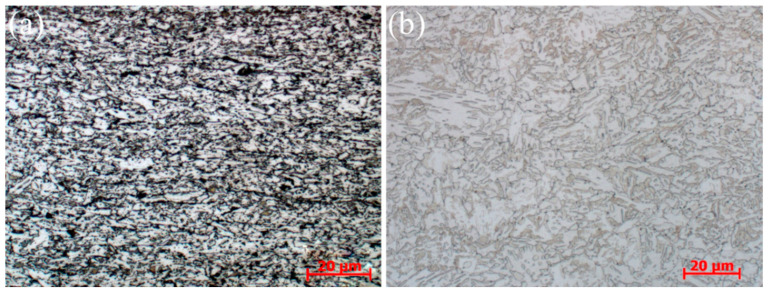
(**a**) As-received sample; (**b**) Sample after austenitizing and furnace cooling.

The experimental steel was received from Magang (Group) Holding Company Ltd. (Maanshan, China), in the form of hot rolled DP steel sheets, and its chemical composition is shown in Table 1. It consists of ferrite and pearlite, as shown in Figure 1a. The plates were cut into several small samples of 100 mm × 50 mm × 2.6 mm. The specimens were first austenitized at 900 °C for 30 min and subsequently cooled in the furnace to obtain a uniform ferrite and pearlite microstructure (Figure 1b). 

**Figure 2:**
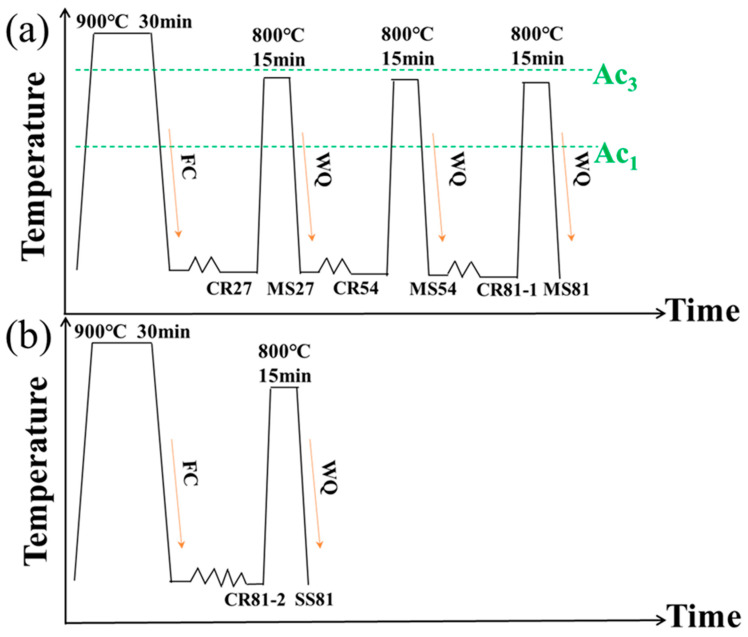
Schematic diagram of (**a**) multi-step and (**b**) single-step rolling and annealing process. Ae_1_: starting temperature of pearlite to austenite transformation during heating; Ae_3_: ending temperature of transformation to austenite after heating.

The rolling process was performed in a cold rolling mill with the roller diameter of 180 mm. Figure 2 illustrates the processing route and marks the names of samples at each stage. The experimental steel was first held at 900 °C for 30 min to fully complete austenitization, followed by cooling within the furnace to obtain a ferrite/pearlite initial structure. The MS process was deformed in a single pass at 27% of the total thickness, and then the samples were held at 800 °C for 15 min. Water quenching was performed to obtain the dual-phase structure. This process was cycled three times. The SS process, on the other hand, performed a single deformation of 81%, followed by holding at 800 °C for 15 min, and a final water quenching. 

### 2.2. Microstructure Characterization

Following etching by the nital solutions, the microstructures were observed using a Zeiss Axiom Observe DIM optical microscope (Oberkochen, Germany). SEM samples were prepared using standard mechanical grinding and polishing procedures. The accelerating voltage was 15 V. Quantitative SEM microstructure evaluation was performed using image analysis techniques to estimate the martensite volume fraction, considering at least 5 images for each specimen. SEM observations were performed in FEI Quanta200 FEG (FEI, Eindhoven, The Netherlands). The average particle size was measured using the intercept method according to ASTM E112-96 [16], considering at least 100 grains.

### 2.3. Tensile Test

Tensile testing was performed using a SANS-CMT5305 testing machine (MTS, Eden Prairie, MN, USA). The gauge length of the tensile sample was 25 mm and the nominal strain rate was 6 × 10^−4^/s. Each tensile specimen test was repeated 3 times to ensure the authenticity of the results. Crussard–Jaoul analysis was used to describe work hardening in the tensile deformation of DP steel [17].

## 3. Results and Discussion

### 3.1. Microstructures

**Figure 3:**
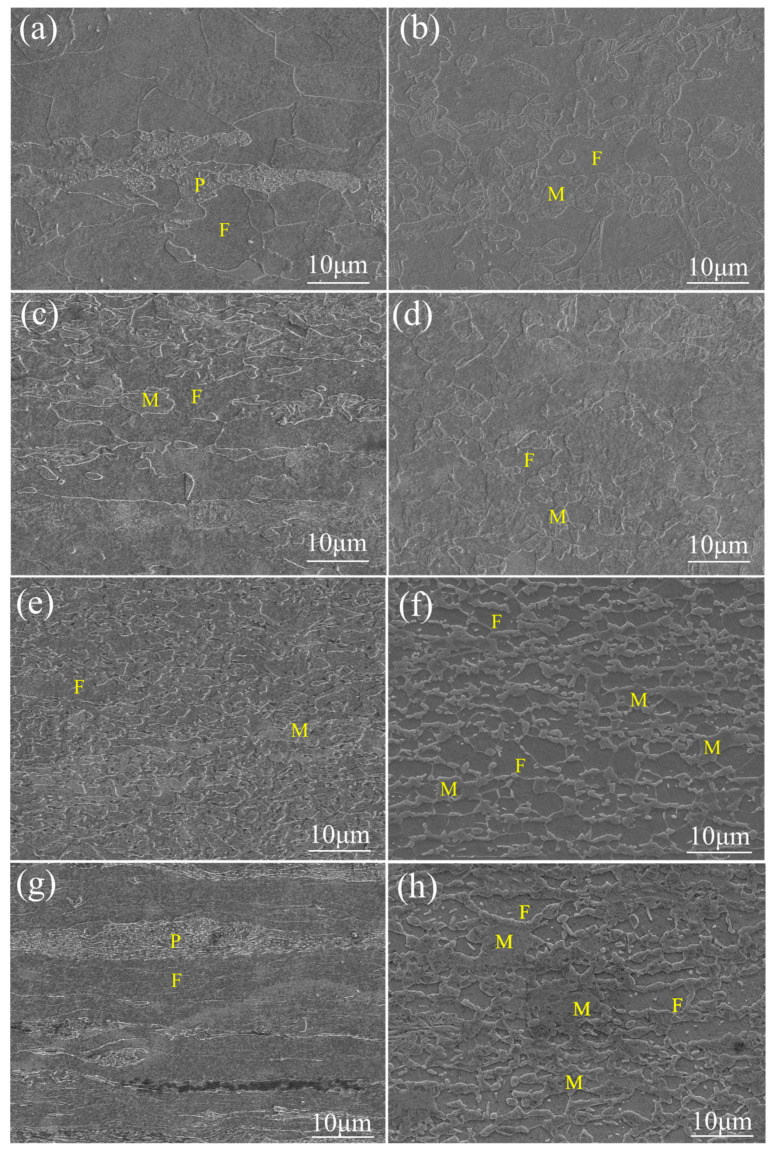
Microstructure of (**a**) CR27; (**b**) MS27; (**c**) CR54; (**d**) MS54; (**e**) CR81-1; (**f**) MS81; (**g**) CR81-2; and (**h**) SS81, M (martensite), F (ferrite), P (pearlite).

SEM micrographs of DP steel obtained using the two different processing routes are shown in Figure 3, where (a, c, e, g) is the rolling state and (b, d, f, h) is the annealing state. 

#### 3.1.1. Comparison of Microstructure after Processing using Different Routes

The microstructures after multi-step (Figure 3f) and single-step treatment (Figure 3h) show a significant difference. The ferrite matrix of MS81 is completely recrystallized and the ferrite grains are equiaxed and uniform in size. The martensite grains are located at the ferrite grain boundaries and are distributed in chains, and a small portion of martensite is located within the ferrite grain, which has a more uniform spatial distribution compared with SS81. The martensite grains of SS81 are mainly located at the ferrite grain boundaries and are aligned in bands along the rolling direction (RD), with the ferrite showing fine striations.

**Figure 4:**
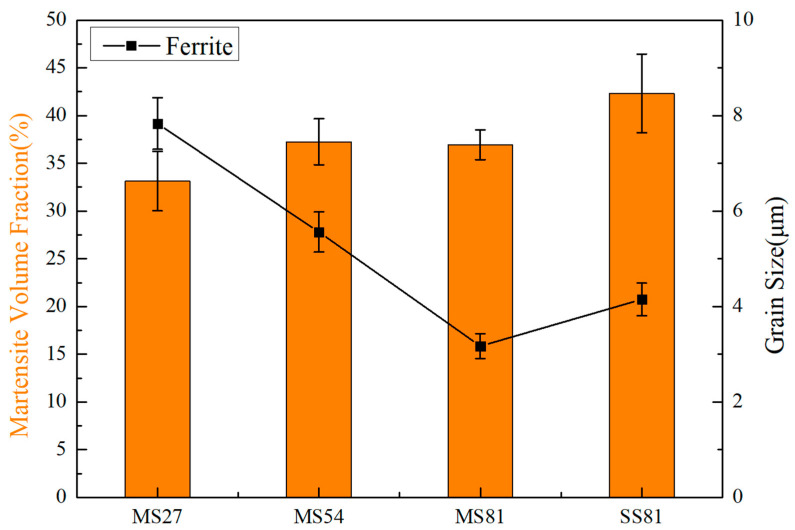
Ferrite grain size and volume fraction of martensite in MS27, MS54, MS81, and SS81.

**Figure 5:**
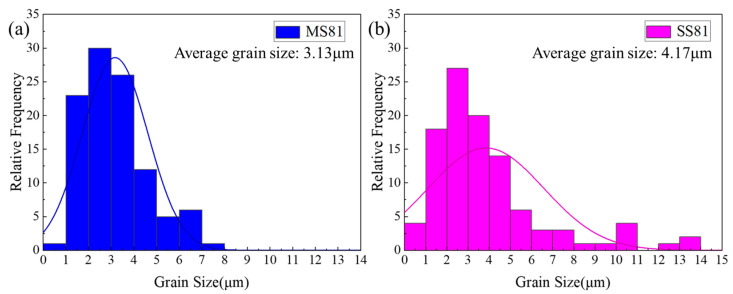
Ferrite grain distribution in (**a**) MS81 and (**b**) SS81.

Figure 4 shows the ferrite grain size and martensite volume fraction for each annealed state. The qualitative description of ferrite grain size, martensite volume fraction, and mechanical property parameters are shown in Table 2. With the increase in deformation during the multi-step cycle rolling and annealing process, the grain size of ferrite decreased. The martensite volume fraction of MS81 is slightly smaller than that of SS81. Figure 5 shows the ferrite grain size distribution of MS81 and SS81. It was found that the average grain size of MS81 (3.13 μm) and SS81 (4.17 μm) differed by about 1 μm. Nevertheless, the grain size distribution of the two different process routes is significantly different.

#### 3.1.2. Microstructure Evolution of Two Different Processing Routes

Figure 3a,b shows the first cycle process. It is clear that the microstructure completes the transformation from pearlite/ferrite to martensite/ferrite; at this point, martensite is mainly present in the form of islands. Figure 3c,d shows the second cycle process. Both ferrite and martensite are striped in the rolling state, and some broken martensite particles are present. In the annealing state, there is a significant refinement of the grains, and the martensite can already be seen in the form of a chain-like distribution. Figure 3e,f shows the third cycle process. At this point, the striped distribution is more obvious. After annealing, the grains are further refined and the martensite shows a clear chain-like distribution.

Figure 3g shows the rolling state of the SS process, with severe deformation of ferrite and pearlite in elongated bands. Figure 3h shows the annealed state, where the martensite shows an aggregated island-like distribution.

During annealing, austenite nucleation occurs, first from the aggregated pearlite, and second, along the ferrite grain boundaries. During the MS process, the martensite will be further broken after cold rolling during each cycle process. Meanwhile, deformation could provide more nucleation points for austenite and increase the deformation storage energy, thus leading to grain refinement and promoting the formation of chain-like martensite distribution.

### 3.2. Tensile Properties

**Figure 6:**
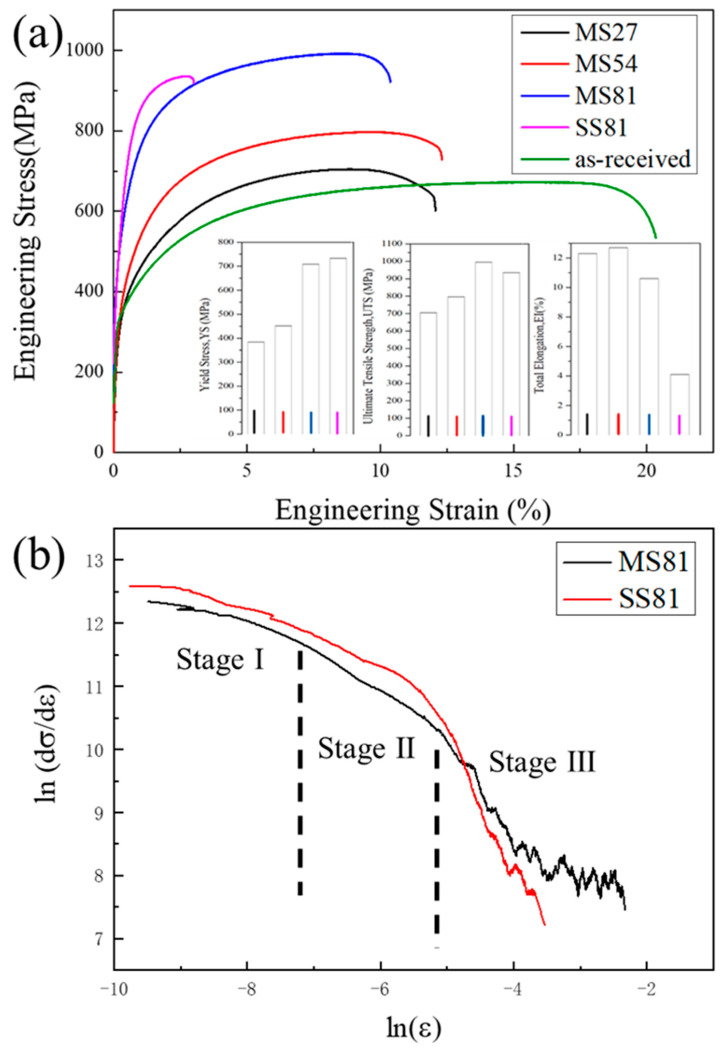
(**a**) Engineering stress–strain curves of MS27, MS54, MS81, and SS81, and (**b**) differential C-J plots of ln(dσ/dε) versus ln(ε) for MS27, MS54, MS81, and SS81.

Figure 6a shows the engineering stress–strain curves of MS27, MS54, MS81, and SS81. The detailed values of the mechanical properties are listed in Table 2. The tensile curves show the typical characteristics of DP steel: no obvious yield point, continuous yield, and high initial strain hardening rate. 

After single-step treatment, DP steel obtained the yield strength (YS) of 733 MPa and ultimate tensile strength (UTS) of 935 MPa, with a relatively high yield ratio and low ductility (uniform elongation 4.1% and total elongation 4.7%). After multi-step treatment, the YS decreased and the UTS increased slightly, resulting in a significant increase in yield ratio compared with the single-step treatment. Moreover, uniform elongation and total elongation were significantly improved to 10.6% and 11.5%, respectively.

Both of the two methods achieved a relatively high strength, which is mainly attributed to the grain refinement processed by the pre-IA treatment (cold rolling) [18,19]. The strength of the DP steel is usually estimated according to the mixture rule [20,21]. The martensite volume fraction (*V_M_*) of DP steel after single-step and multi-step treatment was similar. Simultaneously, the grain size of ferrite after two different routes only changes slightly. Therefore, both the YS and UTS using two different routes show only slight change. However, the ductility of DP steel after the multi-step treatment improved dramatically, which is mainly attributed to the homogenously distributed ferrite/martensite (Figure 3f,h). Compared to the MS process, the SS process was annealed only once and did not undergo intermediate annealing. This means that 81% of the work hardening (Figure 3g) due to deformation cannot be completely eliminated in a single annealing process., which may be one of the reasons for its lower elongation.

The mechanical properties and damage behavior of DP steels were found to be significantly influenced by the martensite distribution [22,23]. In Park’s study [12], the strain ratio of martensite-to-ferrite increased significantly when the martensite in dual phase steel changed from isolated type to chain type, which led to a decrease in the strain distribution between ferrite and martensite. The deformation of ferrite is restricted by the surrounding martensitic structure, which leads to enhanced martensitic deformation. Nouroozi [24] obtained DP steel with a chain distribution using intercritical annealing of cold-rolled martensite, which exhibited high tensile toughness. This was attributed to the fact that chain-like martensite prevented strain localization from propagating to the adjacent ferrite grains. This suggests that a uniformly distributed chain-like martensitic microstructure may improve the strength–ductility balance of DP steel.

The C-J diagram (Figure 6b) of MS81 and SS81 illustrates the three stages of work hardening. Stage I of work hardening is related to the initial density of mobile dislocations in the ferrite, the size of which depends on the size of the ferrite–martensite interface region. Therefore, the expanse of the ferrite–martensite interface can be inferred from the change in the microstructure. In stage II, the ferrite deformation is constrained, and the work hardening behavior is controlled by back stresses and the generation of GNDs (geometrically necessary dislocations) at the interface. The GNDs represent an extra storage of dislocations required to accommodate the lattice curvature that arises whenever there is a non-uniform plastic deformation [25]. The presence of locally aggregated martensite in SS81 can be considered to accelerate the generation of dislocations during yielding, leading to the development of a higher dislocation density. The sharp drop in stage III can be attributed to the deformation of martensite, which is generally accompanied by the slip and dynamic recovery of ferrite at this time. SS81 shows higher work hardening capacity in stages I and II, which is attributed to the localized aggregation of martensitic deformation at the beginning of deformation. MS81 exhibits a higher work hardening capacity in stage III, which is attributed to the homogeneous distribution of martensite that allows for a better synergy between the ferrite and martensite phases during simultaneous deformation.

### 3.3. Fracture Mechanisms

**Figure 7:**
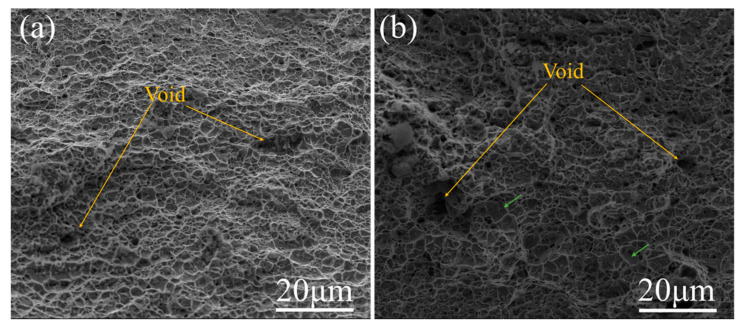
SEM image of the fracture: (**a**) MS81, and (**b**) SS81.

Figure 7 shows the observations of the fracture surfaces of MS81 and SS81. Both samples show a ductile fracture. The fracture of MS81 (Figure 7a) is mainly composed of shallow dimples and a few large dimples. Large dimples may be due to the fracture of larger martensite grains, resulting in larger cavities. SS81 (Figure 7b) contains a large number of voids and some small areas of quasi-cleavage surfaces (identified by green arrows).

As it is well known, the ferrite–martensite interface is the main location for void nucleation [26]. In addition, grain refinement enhances the microstructural resistance to damage [27,28]. In SS81, under the condition of relatively concentrated martensite, when the crack starts to grow, significant elastic energy, stored due to high-strength material deformation, will be released, allowing rapid crack expansion in the destabilized state. Premature martensitic cracking controls the fracture resistance of DP steel. Compared to SS81, MS81 has a more uniform distribution of chain martensite, which leads to a large number of GNDs in the ferrite–martensite interface region at the beginning of deformation and an increasing number of geometrically necessary dislocations as the deformation process continues, which significantly delays the formation of pores and microcracks in the interface region, which is manifested by better ductility.

## 4. Conclusions

In the present work, the evolution of the microstructure and mechanical properties of dual-phase steel after multi-step cyclic rolling and intercritical annealing were studied and simultaneously compared with the results of single-step rolling and annealing processing. The conclusions drawn from the results are as follows:

(1) Fine-grained ferrite and chain-distributed martensite were obtained by a multi-step cyclic rolling and intercritical annealing process. Both the average grain size and martensite volume fraction of the multi-step and single-step treatment are similar. However, the distribution of the ferric and martensitic phases exhibited a significant difference.
(2) Good comprehensive mechanical properties of DP steel (UTS =1 GPa, elongation = 11.5%) were obtained by multi-step cyclic rolling and intercritical annealing. The great improvement of ductility compared to that of the single-step treatment is mainly attributed to the uniform distribution of ferrite–martensite.
(3) The grain size of ferrite decreased and the martensite volume fraction increased with the increase in deformation during the multi-step treatment. The obtained martensite after each annealing was further fractured at each cold rolling stage, and simultaneously, the deformation increased the nucleation points for austenite and stored energy, resulting in grain refinement and the uniform distribution of chain-like martensite.
(4) In the multi-step process, a total of two intermediate anneals were performed, which eliminated the work hardening caused by the rolling process, resulting in a higher elongation than in the single-step process.

## Figures and Tables

**Table 1:** Chemical composition of the studied steel in wt%.

C	Si	Mn	Al	Cr	Mo	P	S	Fe
0.087	0.21	1.99	0.05	0.49	0.18	0.01	0.003	balance

**Table 2:** The average of the measurements and the qualitative description of the components in the microstructure shown in Figure 3.

Sample	YS (MPa)	UTS (MPa)	UE (%)	TE (%)	Yield Ratio
MS27	383.5 ± 15.3	704.3 ± 14.8	12.3 ± 0.3	15.2 ± 0.1	0.51 ± 0.04
MS54	450.7 ± 13.1	796.1 ± 9.3	12.7 ± 0.4	13.9 ± 0.5	0.56 ± 0.02
MS81	708.8 ± 9.5	994.6 ± 22.4	10.6 ± 0.2	11.5 ± 0.6	0.71 ± 0.02
SS81	733.1 ± 6.1	935.4 ± 18.1	4.1 ± 0.4	4.7 ± 1.1	0.78 ± 0.01

## Data Availability

The data that support the findings of this study are available from the corresponding author upon reasonable request.
